# Trend analysis of mortality rates and causes of death in children under 5 years of age in Xuzhou, China from 2016 to 2020

**DOI:** 10.3389/fped.2023.1067293

**Published:** 2023-02-21

**Authors:** Zhiyuan Xu, Haonan Liu, Shuaishuai Zhou, Tiancheng Chen, Chao Meng, Shengli Li, Xianliang Yan, Xiao Liu

**Affiliations:** ^1^ Department of Emergency, The Affiliated Hospital of Xuzhou Medical University, Xuzhou, China; ^2^ Department of Oncology, The Affiliated Hospital of Xuzhou Medical University, Xuzhou, China; ^3^ Department of Cardiovascular, The Affiliated Hospital of Xuzhou Medical University, Xuzhou, China; ^4^ Department of Neurosurgery, The Affiliated Hospital of Xuzhou Medical University, Xuzhou, China; ^5^ Department of Medical Record Statistics, The Affiliated Hospital of Xuzhou Medical University, Xuzhou, China; ^6^ Department of Emergency, Suining County People's Hospital, Xuzhou, China

**Keywords:** mortality, causes of death, children, u5MR, children's health

## Abstract

**Objectives:**

To analyze the trends in mortality and causes of death among children under 5 years of age in Xuzhou, China between 2016 and 2020, in order to protect children's health and provide a basis for formulating child survival, development, and protection strategies.

**Methods:**

A population-based epidemiological study was conducted. Data were obtained from the Xuzhou Center for Disease Control Prevention. We input the data into the excel database and analyzed with SPSS20.0.

**Results:**

There were 1,949 children under 5 years of age died in Xuzhou, The number of deaths from 2016 to 2020 were 573 (29.40%), 577 (29.60%), 371 (19.04%), 334 (17.14%), and 94 (4.82%) respectively, mortality in children showed a downward trend. The number of deaths was relatively high in January (195 cases, 10.01%), February (190 cases, 9.75%), and May (180 cases, 9.24%), while was relatively small in July (147 cases, 7.54%), August (139 cases, 7.13%), and September (118 cases, 6.05%). The leading causes of death (COD) in children under 5 years of age were neonatal suffocation and hypoxia (323 cases, 16.57%). Pizhou (528 cases, 27.09%) showed the highest number of deaths in children under 5 years of age in China, and the Kaifa (25 cases, 1.28%) zone showed the lowest death toll.

**Conclusions:**

Our research suggested that the current strategies for reducing child mortality should prioritize the actions on neonatal deaths and conduct targeted interventions for the main cause.

## Introduction

Child health is commonly regarded as a public priority in every country (1, 2). The Under–Five Mortality Rate (U5MR), which estimates the probability of dying between birth and the fifth birthday (usually expressed per 1,000 live births), is a useful indicator that not only measures the level of child health but also evaluates the overall development of a society (3–5). Significant progress has been made in improving the global child survival rate with the 189 members of the United Nations (UN) adopting the Millennium Development Goals (MDGs) since September 2000 (6–8). In the 21st century, China has made a great effort to improve the survival and development of children and has achieved remarkable success in reducing child mortality, which can be used as a reference for many other low–and middle–income countries (9, 10). The newly launched UN' s Sustainable Development Goals (SDGs) framework calls for an end to preventing deaths of newborns and children under five years of age by 2030 (11). For a populous and diverse country like China, this goal is challenging as the total number of children dying before their fifth birthday is still very large (12). Due to the efforts made by the Chinese government at all levels, the MDGs have been completed in advance. In recent years, with the development of the Chinese economy and improvement of living standards, there has been great progress in improving the health status of children before the establishment of the People's Republic of China. According to a report entitled, “2013 Progress report on China's implementation of the Millennium Development goals”, China is not only gradually completing the healthcare-related MDGs but also expecting to accomplish more national goals (13).

Recently, the U5MR in China has decreased from 61% in the early 1990s to 39.7% in 2000, but it is still much higher than that in some developed countries (13). According to the WHO statistics report on child mortality in 2019, the U5MR of 7.90% in China is higher than that in Britain (4.27%), France (4.46%), Germany (3.81%), and other western developed countries, while it is lower than that in some developing countries, such as Brazil (13.94%), India (34.27%), and Thailand (9.01%) (14). China is still one of the five countries with the highest number of child deaths in the world. As the whole country is taking actions to reduce child mortality, Xuzhou, an important city in the Jiangsu province, is also responding to this call. At the end of 2020, the population aged from 0 to 14 years is 20,311,308, accounting for 22.36% of the overall living population in Xuzhou. Compared with the sixth national census in Xuzhou City, the proportion of the population aged from 0 to 13 years has increased by 4.3% (15).

It is important to regularly update the information regarding the distribution of causes of death (COD) in children for the purpose of policy and research development. In order to investigate the COD, this study aimed to analyze the main results of death surveillance for children under 5 years of age in China from 2016 to 2020, and to provide a basis for the appropriate medical healthcare and policy development.

## Materials and methods

### Patient and public involvement

There was no patient or public involvement in this study.

### Research object

The research object focused on the cases of death in terms of children under 5 years of age who were registered or who lived in Xuzhou between 2016 and 2020. Their mothers experienced a 28-week gestational period and presented one of the four life indicators, including heartbeat, breathing, umbilical cord pulsation, and voluntary muscle contraction after delivery.

### Material collection

Infant death data were transferred to the relevant medical units from the Xuzhou Center for disease control and prevention every month, followed by submission of a verification and supplementary report within 5 days. The surveillance and supervision of COD were performed twice a year and an investigation was carried out three times a year aiming at the failure of reporting COD covering all streets in the district. The survey included information about the birth and death of the resident population per household in the sampling area and ensured integrity of the reported cases of death in the whole region. After completion of the card, the case of death was reported to an online system called the “population death information registration and management system” located in the Chinese Center for Disease Control and Prevention and its Xuzhou division was responsible for checking, counting, and quality control. Since the cases of death in children under 5 years of age were derived from the Xuzhou center, no further ethical approval was required for this study.

### Exclusion and inclusion criteria

Surveillance data between 2016 and 2020 were obtained from the Xuzhou Under-5 Mortality Rate Surveillance Network and Xuzhou Center for Disease Control and Prevention, which covered all children under 5 years of age in 11 districts, including Quanshan, Gulou, Yunlong, Kaifa, Jiawang, Fengxian, Peixian, Tongshan, Xinyi, Pizhou, and Suining. Incomplete files (without COD, or age, or time of death, or native place) were removed, a total of 1949 cases of death in children were collected.

### COD

Clinical diagnosis was used for determining the COD with disease names identified by practical pediatrics. The COD were presented according to the main classes of the 10th Revision of the International Statistical Classification of Diseases and Related Health Problems (16).

### Statistical method

SPSS20.0 and Excel software were used in this study, and data characteristics were calculated and presented in the form of tabulation and graphics. The statistically relevant data of all variables were described in the survey, and they mainly included the data frequency analysis, centralized trend analysis, dispersion analysis, distribution, and some basic statistical graphics.

## Results

**Figure 1:**
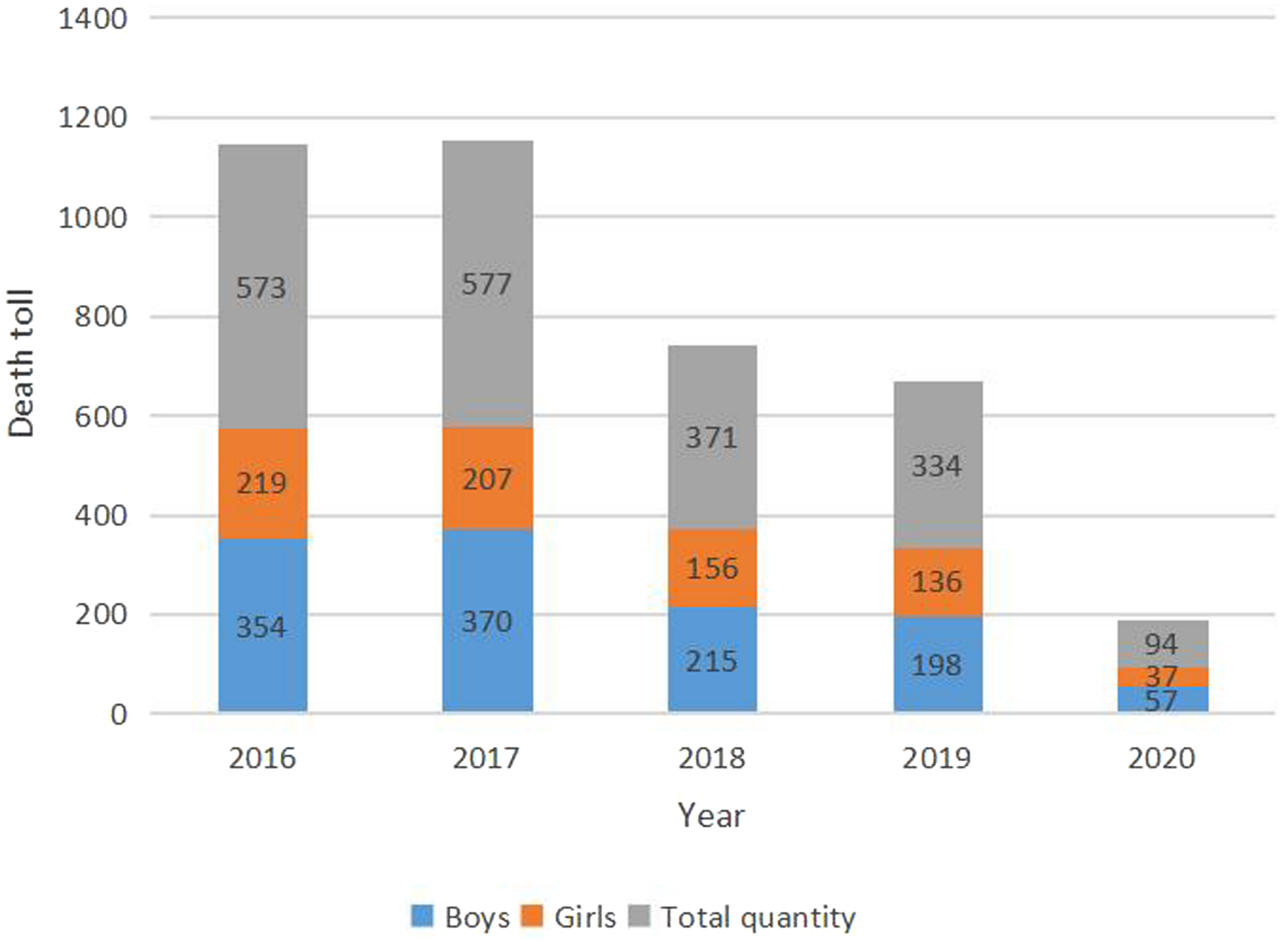
Trends of mortality rates (per 1,000 live births) in China during 2016–2020 for children under 5 years of age.

Figure 1 showed that the number of deaths in these five years, from 2016 to 2020, were 573 (29.40%), 577 (29.60%), 371 (19.04%), 334 (17.14%), and 94 (4.82%) respectively. Compared to the child mortality rate in 2016, the rate in 2020 was decreased by 18.81% and it was found that the number of cases of death in male children was higher than that in female children every year.

**Figure 2:**
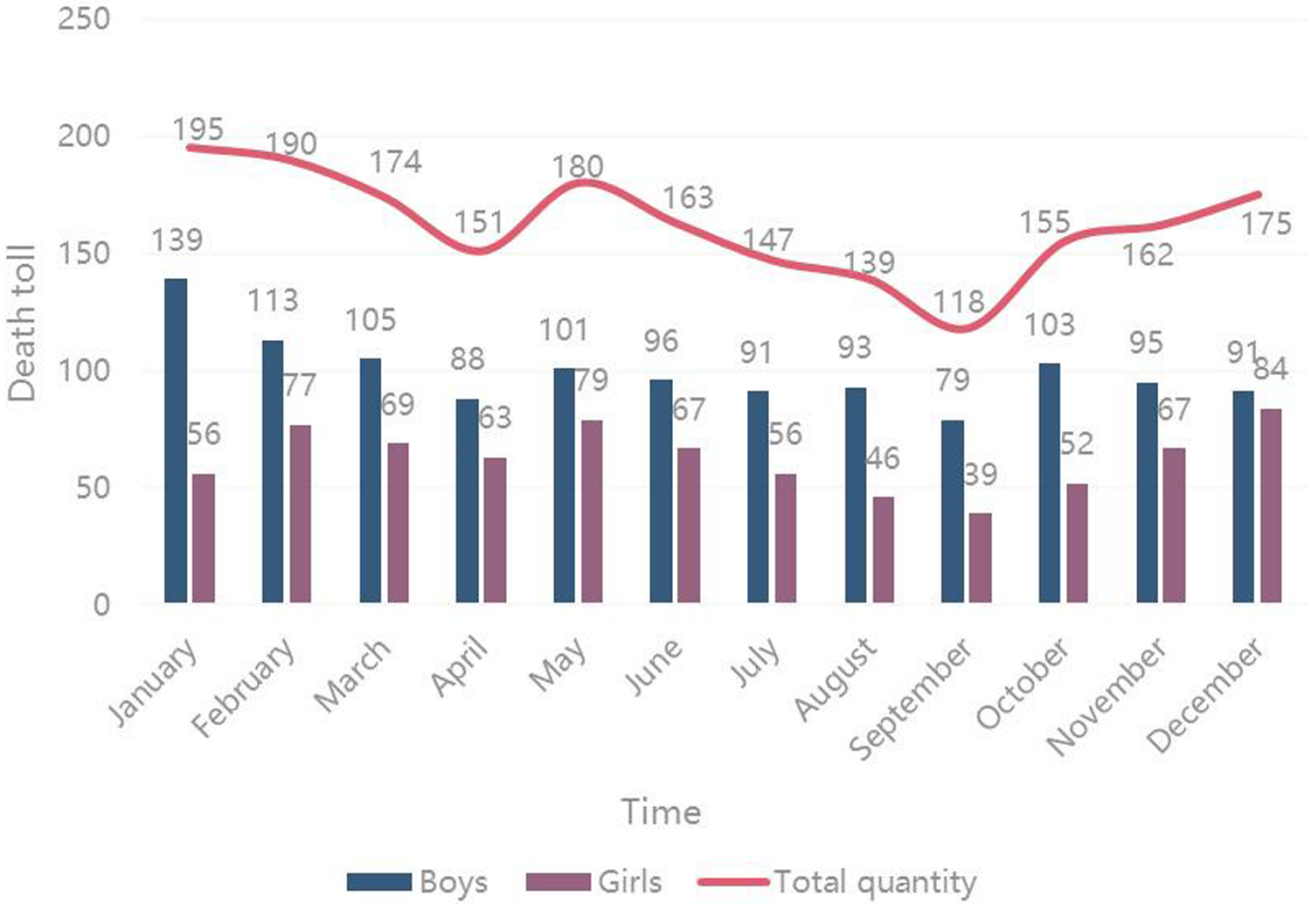
Time distribution of death for children under 5 years of age.

The trend for cases of death per month was observed, and the results showed that high mortality in January, February, and May, while fewer cases of death occurred in April, July, and September. The number of deaths was highest in January and lowest in September. The number of deaths in male children was higher than that in female children every month (Figure 2).

The COD were classified and summarized according to the International Classification of Diseases 10th Edition (ICD10) and sorted according to the number of deaths. The top ten COD were listed in Table 1, and they were neonatal suffocation and hypoxia (accounting for 16.57% of deaths), congenital heart disease (accounting for 12.52% of deaths), pneumonia (accounting for 11.29% of deaths), tracheal obstruction and mechanical suffocation (accounting for 6.82% of deaths), sepsis (accounting for 1.44% of deaths), and nervous system disease (accounting for 0.82% of deaths).

**Table 1:** The main COD and composition.

	Death toll	Percentage (%)
Neonatal suffocation and hypoxia	323	16.57
Congenital heart disease	244	12.52
Unripe and defective infants	220	11.29
Tracheal obstruction and mechanical suffocation	133	6.82
Drowning	89	4.57
Traffic accident	76	3.90
Congenital pulmonary disease	40	2.05
Digestive system disease	39	2.00
Sepsis	28	1.44
Nervous system disease	16	0.82
Other diseases	741	38.02
Total deaths	1,949	100.00

**Notice**: Classify the COD based on ICD10 (International Classification of Diseases 10th Edition).

**Figure 3:**
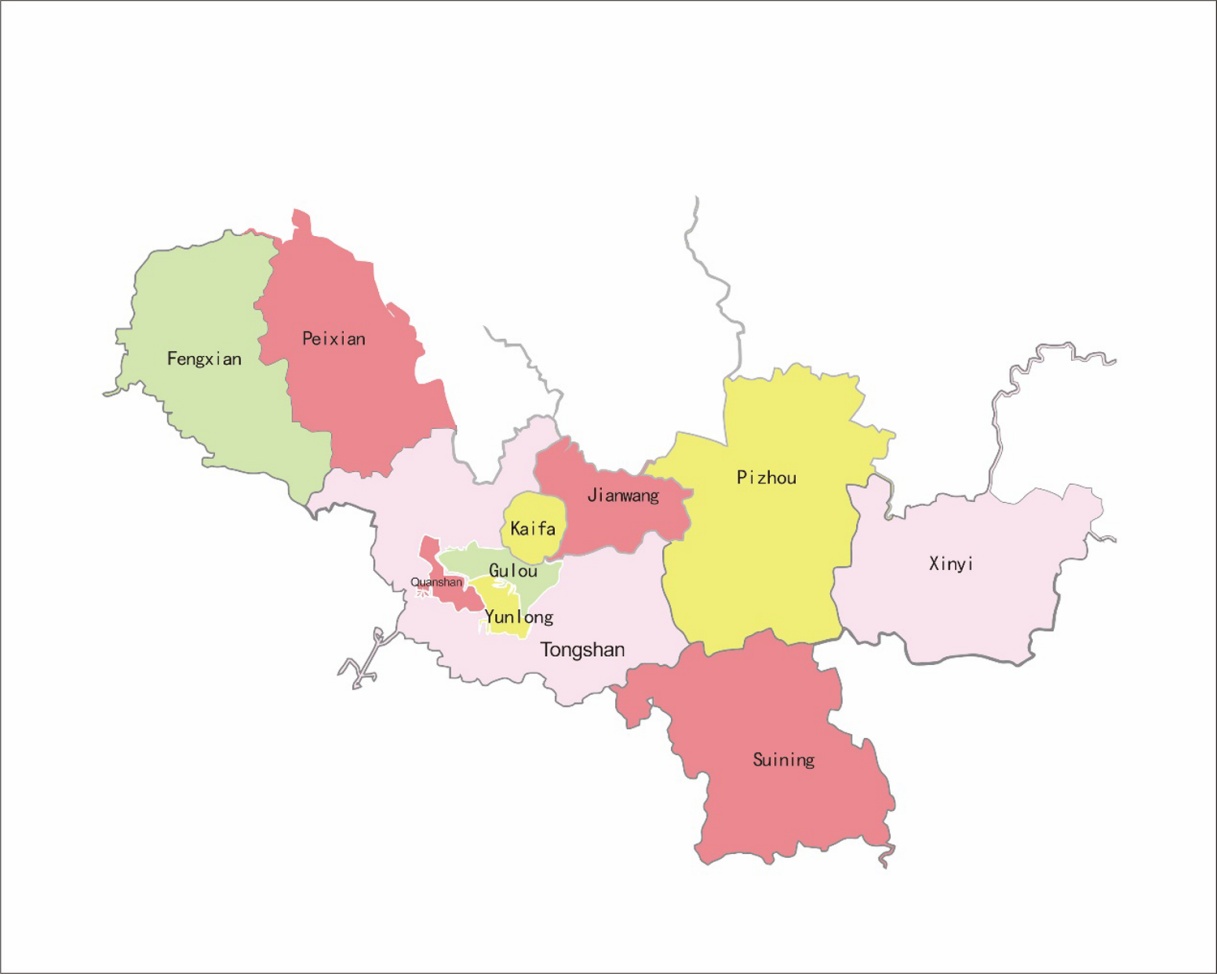
Map of Xuzhou city.

Xuzhou is not only a prefecture-level city in China, but also a famous national historical and cultural city. With the Beijing Hangzhou Grand Canal passing through Xuzhou, transportation is very convenient and thus the city is known as the thoroughfare of five provinces. The regions and locations of each district in Xuzhou are as follows: the first is Pizhou, which covers a total area of 2,088 km^2^ and governs 24 towns; the second is Tongshan with a total area of 2,003.98 km^2^ and covering 11 streets and 17 towns; the third is Peixian County, with a total area of 1,806 km^2^, which governs 4 streets and 13 towns; the fourth is Suining with a total area of 1,769 km^2^ and governing 3 streets and 15 towns; the fifth is Xinyi with a total area of 1,616 km^2^; the sixth is Fengxian with a total area of 1,450.2 km^2^ and governing 3 streets and 12 towns; the seventh is Jiawang with a total area of 690 km^2^ and governing 7 streets and 5 towns; the eighth is Kaifa with a total area of 293.6 km^2^; the ninth is Yunlong, which covers a total area of 118 km^2^ and governs 9 streets; the tenth is Quanshan with a total area of 100 km^2^ and a total of 14 streets; the last one is Gulou which covers a total area of 90.8 km^2^ and governs 9 streets. As shown in Figure 3.

**Figure 4:**
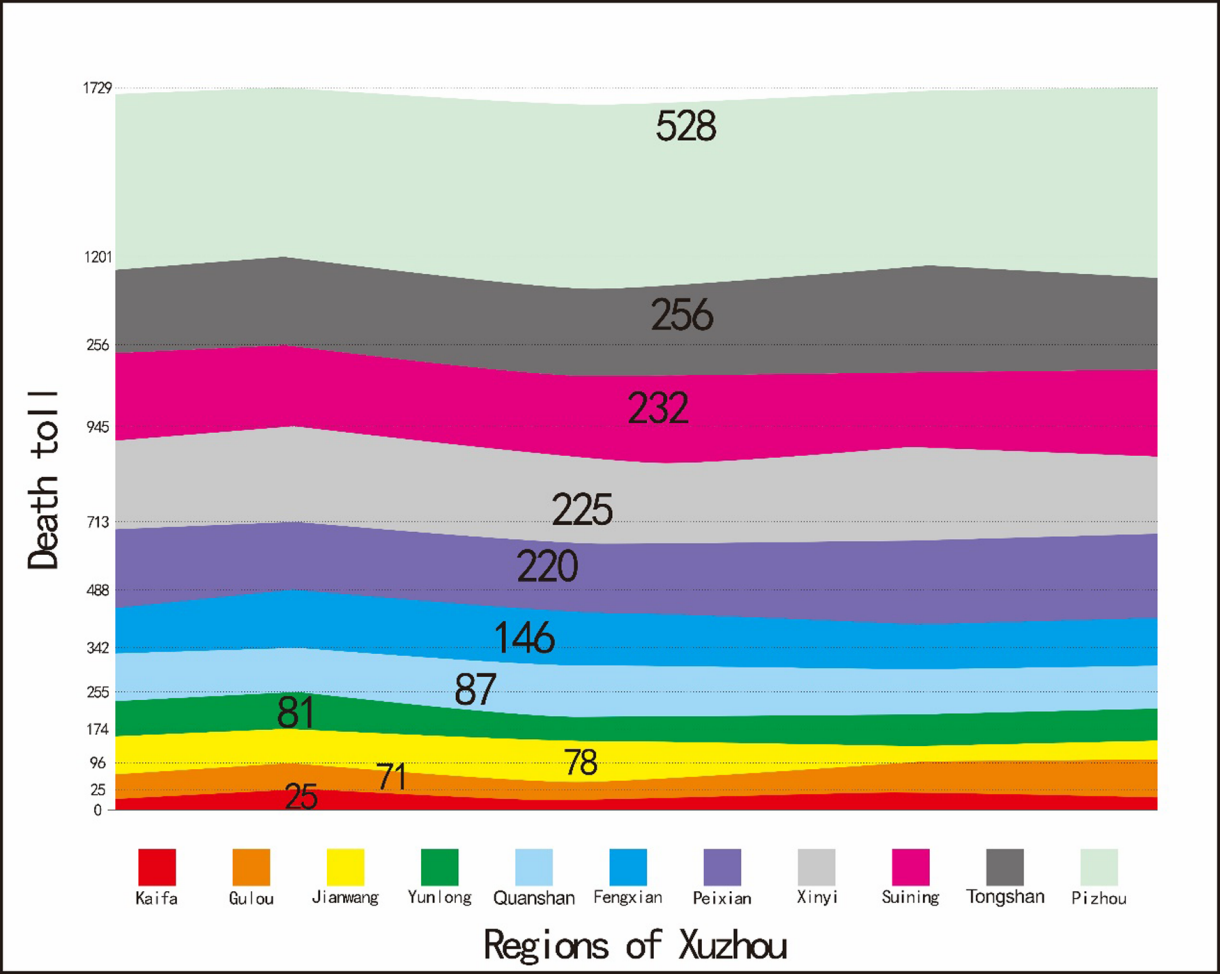
Regional distributions of death for children under 5 years of age in Xuzhou.

There are three distributions of diseases in epidemiology, including population distribution, time distribution, and regional distribution. The regional distribution of cases of death in children under 5 years of age showed in Figure 4. The results revealed that Pizhou showed the highest number of cases of death in children under 5 years of age, followed by Tongshan, Suining, Xinyi, and Peixian. Kaifa zone presented the lowest number of cases of death; and Gulou, Jiawang, and Yunlong also showed a relatively low number of cases of death.

## Discussion

With the socio- economic development, the living standard has improved and child mortality has declined. However, many current challenges remain (17–19). Child mortality can be clearly understood when the COD are reliably sorted. Some causes may be more likely to reduce than others since the combination of causes varies remarkably in cases of different times and ages (20, 21). The primary aim of this study was to estimate the proportions of different COD in children under 5 years of age, and the spectrum of COD in children in Xuzhou city, China from 2016 to 2020 was successfully defined in this study. The relationship between different COD in children under 5 years of age has proven to be a useful approach to predict the main COD based on the overall mortality.

Overall, the trend of the mortality rate for children under 5 years of age was decreased from 2016 to 2020, while it was slightly increased in 2017. However, in 2020, this downward trend was significantly strengthened. Due to medical reforms in China, medical and health work has made great progress in the past few years. The number of deaths in children under 5 years of age was decreased every year with the enhancement of child survival. The presence of COVID-19 virus may also reduce the mortality rate of children under five years old. In this severe epidemic environment, parents pay more attention to their children's health. They put masks on their children and reduce the frequency of egress, which greatly reduced the chance of contact with COVID-19 virus. Winter was a season of high mortality for children under five years old, the lowest mortality was in September. Based on the above analysis, it can be concluded that cold weather and low temperatures may cause maladjustment to the outside environment and that the lack of measures to keep warm may cause the death of children. Meanwhile, September is a transitional period of the year, and the adaptable climate and temperature are conducive to the survival of children. September is the beginning of the new term of kindergarten in china, and children will go to school with their teachers or relatives, which will provide some protection for children. Some studies have shown that it is possible to reduce perinatal mortality after birth by keeping the baby warm on time (22). Advanced maternal age has been proven to be a risk factor for many disorders in children under 5 years of age, such as prematurity and congenital abnormalities (23–25). However, it has also been found that excessive warmth should be avoided due to drought and insufficient warmth may cause colds and other diseases (26). Both these conditions will threaten the survival of newborns. Therefore, the governments and medical units should emphasize the topic of children's health at the turn of the season, especially in winter, to attract the attention of parents. It is extremely important to give a suitable temperature around the newborns.

Neonatal suffocation and hypoxia was the most important COD, particularly in newborns or infants. Disorders of gas exchange due to various causes before birth, at birth, and after birth will cause suffocation and hypoxia. It is not possible for newborns to establish normal spontaneous breathing after birth. Therefore, any factor that reduces the oxygen concentration in the fetus and newborn can cause hypoxia and asphyxia, which is derived from the gestational period, but it mostly occurs after the beginning of labor. If hypoxia is serious and occurs earlier, the fetus can die in the womb. Doctors and parents should take good care of the perinatal period. Regular physical check-ups are necessary for mothers and newborns with high-risk pregnancies. Doctors can tell whether the baby is lack of oxygen by the mixing of amniotic fluid and the blood gas analysis of the fetus. If the baby has a high degree of hypoxia, doctors and nurses should prepare emergency measures after delivery. It is necessary to monitor the hypoxia condition of children from parturition to delivery. If a gravida has an accident or there is high-risk factor for pregnancy and delivery, they should go to a special hospital in time. The hospital should have complete facilities and well-trained Midwives. Congenital heart disease was the second leading COD, with evidence showing that genetic factors influence this COD. The prevalence of congenital heart disease in parents and their children is higher than that in the general population. Most congenital heart diseases caused by polygenic defects, and some of them can be caused by a single genetic defect or chromosomal aberrations. Unripe and defective infants are the third leading COD in girls under 5 years of age. In addition, tracheal obstruction and mechanical suffocation, drowning, and traffic accidents are also important COD in children under 5 years of age. As the children grow up, parents gradually pay less attention to their care; thus, allowing their children to play with water but forget the time period and this eventually results in accidental drowning. Furthermore, children are prone to develop mechanical asphyxia due to food items (e.g., dates, fish, and bones) getting lodged in the trachea and incorrect sleeping postures. Through the above analysis, strengthening perinatal health care and improving the nutrition and health status of pregnant women are very important for preventing and reducing the fetal and infant mortality rate.

The area with the highest death toll was Pizhou, which is the largest district in Xuzhou. Pizhou was a famous place for strategists in ancient times. Pizhou, with developed transportation, is adjacent to coastal cities in the East and Xuzhou urban area in the West. From the seventh national census bulletin of Xuzhou until the end of November 1, 2020, the permanent resident population of Pizhou accounts for 16.0% of the population in Xuzhou. There is a large proportion of deaths among children in regions, such as Tongshan, Suining, Xinyi and Peixian for similar above-mentioned reasons. The area with the lowest number of deaths among children was the Kaifa zone, which is in the stage of construction and development. Compared with other areas, transportation is inconvenient and the population is relatively small. Other areas, such as Gulou, Jiawang, Yunlong, and parts of Xuzhou City, are very close to the hospitals and have convenient transportation. As a result, the number of child deaths is relatively low. Therefore, in order to allow children to receive effective treatment before death and save their lives, the provincial government of Xuzhou should popularize disease knowledge and actively publicize the essentials of on-the-spot rescue for common emergency diseases. The economic and transportation facilities in the surrounding areas should be strengthened, especially focusing on areas far away from hospitals and their medical service level.

Compared with the infant mortality rates in China (38 deaths per 1,000 live births) provided by UNICEF, this rate is considerably lower than that in some Asian countries (e.g., India with 71 deaths per 1,000 live births and Mongolia with 105 deaths per 1,000 live births) (27). However, it is higher than that in Korea (6 deaths per 1,000 live births) and Japan (4 deaths per 1,000 live births) (26). In the last two decades, the infant mortality rates in urban areas in 2013 were lower than those in the Republic of Korea (3 deaths per 1,000 live births), and similar to that in Japan (2 deaths per 1,000 live births) (27). These substantial changes may be attributed to the efforts made by the Chinese government. Additionally, the National Health Commission of the People's Republic of China has established a nationwide mortality surveillance network that collects children's health information from the representative population samples to obtain accurate mortality estimates among Chinese newborns, infants, and children under the age of 14 years (28).

In summary, the results of this study are very important for decision makers to understand the dynamics of infants and children under 5 years of age. By understanding the relevant epidemiological characteristics in Xuzhou, policymakers can develop corresponding measures to reduce child mortality. Similarly, not only Xuzhou but also the similar cities can refer to our results to develop protection measures.

## Data Availability

The raw data supporting the conclusions of this article will be made available by the authors, without undue reservation.
